# Violations of the International Code of Marketing of Breast-milk Substitutes: a multi-country analysis

**DOI:** 10.1186/s12889-022-14503-z

**Published:** 2022-12-13

**Authors:** C. K. Lutter, S. Hernández-Cordero, L. Grummer-Strawn, V. Lara-Mejía, A. L. Lozada-Tequeanes

**Affiliations:** 1 International Development Group, Food Security and Agriculture, RTI International, 701 13Th Street NW, Suite 750, Washington, DC 20005-3967 USA; 2 Research Center for Equitable Development EQUIDE, Universidad Iberoamericana, Prolongación Paseo de La Reforma 880, Lomas de Santa Fe, 01219 Mexico City, Mexico; 3 Department of Nutrition and Food Safety, World Health Organization, Geneva, Switzerland; 4 Nutrition and Health Research Center, National Institute of Public Health, Santa Maria Ahuacatitlán, 62100 Cuernavaca, Morelos, Mexico

**Keywords:** International Code of Marketing of Breast-milk Substitutes, Breastfeeding, Child feeding, Child nutrition

## Abstract

The marketing of formula milk as a substitute for breast milk continues to be ubiquitous and multifaceted despite passage by the World Health Assembly of the International Code of Marketing of Breast-milk Substitutes (the Code) in 1981. In this paper, we summarized reports of the Code violations from eight studies using the WHO/UNICEF NetCode protocol. Among 3,124 pregnant women and mothers with young children, in eight countries, 64% reported exposure to promotion of products covered under the Code in the previous 6 months, primarily from advertisements seen outside of health facilities (62%). Nearly 20% of mothers with an infant < 6 months reported that a health care provider had advised them to feed their child food or drink other than breast milk, and 21% of providers reported contact with a representative of a formula company in the previous 6 months to distribute promotional materials, samples, or free supplies (range 2%–53%). Of the 389 retail stores and pharmacies surveyed, promotions were observed in 63% (range 0–100%), and of 1,206 labels and inserts of products reviewed, nearly half included health and/or nutrition claims (range 0–100%). A strong, though non-significant, linear relationship between the composite violations score and quality of Code legislation was found; countries with the lowest percentage of violations had the strongest Code legislation. In Latin America, over 50% of health care providers reported no knowledge of the Code, and 50% reported no knowledge of national legislation. Our study highlights three key facts: 1) the marketing of BMS is ubiquitous and multifaceted, 2) the high variability of promotion across countries generally reflects the comprehensiveness of Code legislation, and 3) health care providers have poor knowledge of the Code and national legislation.

**Supplementary Information:**

The online version contains supplementary material available at 10.1186/s12889-022-14503-z.

## Introduction

Marketing of formula milk as a substitute for breast milk is ubiquitous and multifaceted. More than 80 years after the promotion of milk-based formulas resulting in malnutrition and mortality among infants and young children in low- and middle-income countries was first highlighted [1] and 50 years after the term “commerciogenic malnutrition” was coined [2], the marketing tactics of milk formula companies have only grown more sophisticated and manipulative.

In 1979 the World Health Organization (WHO) convened the first meeting on infant and young child feeding, which was attended by about 150 representatives of governments, non-governmental organizations (NGO), academics, and the infant food industry [3]. The meeting called for an International Code of Marketing of Breast-milk Substitutes (the Code) to be developed by the United Nations Children’s Fund (UNICEF) and WHO. Two years later, the Code was endorsed by the World Health Assembly (WHA) [4] and in 2021 celebrated its 40th anniversary [5]. To date, some provisions of the Code have been adopted in 144 (74%) WHO member countries [6].

The Code recognizes that pregnant women, mothers, and their families and health care providers are susceptible to direct and indirect marketing strategies. Consisting of 11 articles, it outlines the responsibilities of governments, health care systems, health care providers, and companies that market or manufacture breast-milk substitutes (BMS) for taking measures to protect breastfeeding (BF). The Code defines a BMS as any food being marketed or otherwise represented as a partial or total replacement for breast milk, whether or not suitable for that purpose [4]. Together with 19 subsequent relevant resolutions or decisions endorsed by the WHA, the Code represents the collective will of the member states and carries substantial political and moral weight (Nutrition and Food Safety (who.int). However, without enforceable legislation and investment to support monitoring, it will not have full effect [6]. Because of lacking or weak legislation and enforcement, violations frequently occur in most countries. Marketing by the large and growing BMS industry, which now promotes follow-up formulas (FUF) and growing-up milks (GUM) deemed unnecessary by the WHO and numerous professional societies [7], continues to undermine confidence in and the perceived value of BF [8, 9].

The risks of not BF for infant and young child health and development and for maternal health are significant [10, 11], and the economic costs of not BF are well-documented [12]. Thus, the Code is as relevant today as it was 40 years ago when endorsed by the WHA.

To improve global monitoring and enforcement of the Code, the Network for International Monitoring of the Code (NetCode) was founded by the WHO and UNICEF in 2014 and includes eight NGOs, including The International Action Baby Food Action Network (IBFAN), La Leche League International, and Helen Keller International, among others. The NetCode partnership published two research protocols, one for continual assessment and one for periodic assessment of adherence to the Code and/or national legislation [13]. To date, monitoring based on the NetCode periodic assessment protocol has been implemented by 13 countries. The purpose of this paper is to report on the results of eight of these countries.

## Methods

The NetCode protocols focus on promotion of any product that is within the scope of the Code, including milk products specifically targeted for children < 3 years (infant formula, FUF, and GUM), foods or liquids targeted for infants < 6 months, and feeding bottles and teats. As noted in the protocol for periodic assessment, "Periodic assessment is essential to measure the level of compliance with the Code and national laws, assess trends in the marketing of breast-milk substitutes, and prioritize key issues to be addressed with strengthened legislation, interventions, and funding" [13]. The objectives of the protocol are to:

- detect violations of the national laws and/or the Code;
- document and report such violations;
- investigate and validate whether the reported activities are indeed violations;
- activate an enforcement mechanism that would stop such violations and deter future violations; and
- hold manufacturers, distributors, retail outlets, the health care system and health care workers to account for their breeches of national laws and/or the Code

The protocol for periodic assessment lays out procedures for data collection from mothers of children 0–23 months of age, health care providers, and primary health care and maternity facilities. It also lays out procedures for data collection in brick-and-mortar retail stores, such as pharmacies and grocery stores, on television, and through the internet and social media. The protocol includes a standard questionnaire for face-to-face interviews with mothers and health care providers (with a recall period of the previous 6 months), a set of procedures for evaluating television and internet advertisements, tools for observing and recording promotions in retail stores and primary health care facilities, and a checklist for evaluating information on product labels.

Our paper consolidates results from use of the protocol for periodic assessment in eight countries: five in Latin America (Chile, Ecuador, Mexico, Panama, and Uruguay), one in Africa (Nigeria), and two in Asia (the Philippines and Thailand) [14–21]. According to the World Bank, of these countries, two are classified as upper income (Chile and Uruguay), four are classified as upper-middle income (Ecuador, Mexico, Panama, and Thailand) and two are classified as lower-middle income (Nigeria and the Philippines) (Supplementary Table 1) [22]. Among the countries, three had some provisions included, two were moderately aligned, and three were substantially aligned. Total scores ranged from 29 (Chile) to 85 (Philippines).

Data were extracted by two coauthors (VLM and ALLT). Whenever there was a discrepancy, a third coauthor reviewed the data (SHC).

We report on all aspects of the protocol, though acknowledge that documentation of promotion of BMS through internet advertisements and social media is likely to be underestimated because of the challenges associated with collection of such data.

All studies were conducted between 2016 and 2020. The authors of this paper were involved in the studies carried out in Ecuador, Chile, and Mexico. Approval from the national or institutional ethics committees was obtained for each country. All participating mothers and health care providers signed a letter of informed consent. Each country made some modifications to the protocol to address specific national concerns. As such, the sample sizes for the different instruments of the protocol differ across countries, and in some countries, questionnaires were abbreviated. For example, while the protocol requires data collection only in the largest city of the country, Ecuador and Mexico selected two cities. In Chile, private health clinics were excluded from the sample; however, maternity facilities were included. The studies in Nigeria, the Philippines, and Thailand were conducted primarily for the purpose of assessing infant formula manufacturers’ compliance with the Code and thus used an abbreviated questionnaire for health care providers and mothers. Finally, in Nigeria, the Philippines, Thailand, Panama, and Ecuador mothers’ exposure to promotion or messages from mass media, outside the health facility, was presented using the total number of reports as the denominator rather than the total number of mothers interviewed. For this reason, we could not include this information in our paper. Details of the studies for each country are shown in Supplementary Table 2.

The protocol calls for a random sample of 33 health care facilities providing well-baby care services. In each facility, five mothers with children < 6 months old and five mothers with children > 6 to 23 months old were selected. The sample size of 330 for the mother’s questionnaire was designed to detect a 10% prevalence rate of exposure to BMS promotions within the health care system, with confidence intervals at 95% and a measurement error of ± 5%, assuming a design effect of 2 to account for the cluster design. For each health care facility, between one and three health providers were interviewed using a convenience sample.

Data collection at each point-of-sale location included enumeration of products sold under the scope of the Code. In Chile, Ecuador, Mexico, Panama, and Uruguay, a list of the products encountered was recorded. In Nigeria, the Philippines, and Thailand, a preliminary list of products from an internet search was compiled and refined through confirmation of the ones available in each country.

For the review of labels and inserts, we reviewed products, primarily from milk formulas for children 0 to 36 months and complementary foods, assessing the extent of promotions and compliance of product labels with the Code and national laws.

To examine the association between Code violations and quality of Code legislation, we first estimated a composite violation score for each country as a weighted average of the percent of mothers, health care providers, retail outlets, or product labels that showed violations of the Code. Briefly, exposure to promotions of BMS through health care providers, retail outlets, or product labels were weighted equally, receiving 20 points each, and exposure to promotions reported directly by mothers received twice that weight (40 points). Where multiple types of promotion were assessed within each of these categories, the points were distributed among the different types. The weights are shown in Supplementary Table 3. We then examined the association between the composite violation score and the quality of the country’s Code legislation as presented in the 2022 Status Report of National Implementation of International Code [6]. That report classified countries according to how well their legislation reflects the provisions of the Code on a scale from 0 to 100.

We used Excel (version 16.50) for all analyses. Results were calculated for each country and for all countries.

## Results

Our study included 3,124 women, ranging from 330 to 693 women per site (Table 1). The average age was 27 years. Almost 80% had attended middle school or beyond, and 75% attended public health facilities for maternity and post-partum care. As specified in the protocol, the 3,124 children were about evenly split between those < 6 months and 6 to 23 months.

**Table 1:** Sample characteristics of mothers, health care providers, health care facilities, and retail outlets, by country (%)^a^

	**Chile (2017)**	**Ecuador (2017)**	**Mexico (2016)**	**Nigeria (2018)**	**Panama (2019)**	**Philippines (2021)**	**Thailand (2018)**	**Uruguay (2019)**	**Total**
**Women**									
	*n* = 451	*n* = 330	*n* = 693	*n* = 330	*n* = 330	*n* = 330	*n* = 330	*n* = 330	*n* = 3124
Type of health facility (%)									
Private	0	15.2	24.2	81.8	7.9	21.2	9.0	45.5	24.5
Public^b^	100	84.8	75.8	18.2	89.4	78.8	91.0	54.5	75.3
Other	-	-	-	-	2.7^c^	-	-	-	0.2
Age (years) (Mean ± SD)	28.0 ± 6.0	27.1	25.7 ± 6.4	-	-	-	-	-	26.9
Age of child (%)									
< 6 months	62.3	50.3	51.5	50.9	51.8	50	34.8	45.2	50.3
6–23 months	37.7	49.7	48.4	49.1	48.2	50	65.2	54.8	49.7
Education (%)									
None	-	0.6	0.8	-	0.6	-	-	0	0.6
Elementary school	-	16.3^d^	21.3	-	6.4	-	-	19.0^d^	18.1
Middle school	-	20.3	41.0	-	20.1	-	-	35.0	33.0
High school or technician	-	43.3	26.8	-	41.6	-	-	26.0	31.8
Professional or more	-	19.4	9.8	-	31.3	-	-	20.0	16.5
**Health care providers**									
	*n* = 164	*n* = 66	*n* = 48	*n* = 98	*n* = 107	*n* = 126	*n* = 99	*n* = 154	*n* = 862
Type (%)									
Physician	25.0	62.0	62.5	9.2	32.7	19.8	2.0	25.0	25.8
Nurse	25.0	18.2	22.9	52.0	32.7	30.2	77.8	38.0	37.6
Obstetrician	23.8^e^	7.6	-	6.1^e^	-	40.5^e^	-	20.0	15.2
Other^f^	26.2	12.1	14.5	32.7	34.6	9.5	20.2	17.0	21.4
**Health care facilities**									
	*n* = 39	*n* = 33	*n* = 48	*n* = 33	*n* = 35	*n* = 43	*n* = 33	*n* = 33	*n* = 297
Sector (%)									
Private	0.0	15.2	39.6	81.8	5.7	39.5	9.1	45.5	29.6
Public	100	84.8	60.4	18.2	94.3	60.5	90.9	54.5	70.4
Type (%)									
Primary health center	89.7	69.6	66.6	100	77.1	76.7	84.8	100	82.2
Doctor´s office	-	-	4.2	-	-	-	-	-	0.7
Maternity facility/Hospital	10.3	30.3	29.1	-	22.9	23.3	15.2	-	17.1
**Retail outlets**									
	*n* = 80	*n* = 44	*n* = 51	*n* = 43	*n* = 41	*n* = 43	*n* = 43	*n* = 44	*n* = 389
Type (%)									
Supermarkets	12.5^ g^	11.4	54.9	23.3	24.4^ g^	23.3	23.3	13.6	22.9
Convenience and corner stores^h^	36.2	-	13.8	76.7	75.6	76.7	76.7	-	42.7
Pharmacies^i^	51.3	88.6	31.3	-	-	-	-	79.5	33.7
Department stores	-	-	-	-	-	-	-	6.8	0.7

^a^In parentheses, the year when the study was conducted

^b^Includes maternity health facilities and public primary health facilities

^c^Not specified

^d^Includes incomplete or complete elementary school

^e^Includes midwives

^f^Nutritionist, administrative personnel, department head, director or sub-director of the health facility, and dentist

^g^Includes all large establishments

^h^Convenience store refers to small-medium establishments that sell food products and can be franchises or chain, and corner store refers to local owners, and their establishments are smaller than convenience stores

^i^The reports from Nigeria, the Philippines, and Thailand did not differentiate between convenience stores and pharmacies; therefore, the results are included in the data for convenience and corner stores

Of the health care facilities, 70% were public and 30% were private with large variation among countries. Most were primary health care facilities, followed by maternity facilities and hospitals, and doctor’s offices. A total of 862 health care providers were interviewed, including nurses (38%), general physicians (26%), obstetricians (15%), and others such as nutritionists, administrative personnel, and department heads (21%), with large variation among countries.

For assessments at retail stores and pharmacies, convenience and corner stores were most surveyed (42%), followed by pharmacies (34%), supermarkets (23%), and department stores (1%), with large variation among countries.

Overall, 64% of mothers reported exposure to BMS promotion in the previous 6 months, primarily from advertisements seen outside of health facilities (62%) (Table 2). We observed large variation among the countries, with almost 87% of women in Chile reporting exposure to BMS promotion, followed by Mexico (85%) and Thailand (83%). The lowest reported rates of exposure were observed in Nigeria (18%). Women in Nigeria, the Philippines, and Thailand reported no exposure in health care facilities. Reported promotion to mothers included a gift (e.g., toy, bag, bib, etc.,) (14%) or free sample of any baby milk products such as infant or follow-up formula (7%), with variation among countries. Among the three studies that interviews counties that collected information on maternal report conducted media monitoring, television was the most reported medium for exposure (63%), followed by promotions at retail stores and pharmacies (15%).

**Table 2:** Mothers’ exposure to promotion or messages at the health care facility, mass media, or other source related to any breast-milk substitutes or companies selling these products, by country (%)^a^

	**Chile *(n* = 451)**	**Ecuador *(n* = 330)**	**Mexico (*n* = 693)**	**Nigeria (*n* = 330)**	**Panama (*n* = 330)**	**Philippines (*n* = 330)**	**Thailand (*n* = 330)**	**Uruguay (*n* = 330)**	**Total**^**b**^** (*n* = 3124)**
**Heard and/or seen a promotion or message at**									
Promotion in general	86.7	82.4	84.4	18.0	70.6	43.9	83.0	42.2	63.9
Health facility^c^	3.8	2.7	3.3	-	4.5	-	-	3.2	-
Promotion outside health facilities	82.9	79.7	81.1	18.0	66.1	43.9	83.0	39.0	61.7
Television	58.3	-	69.1	-	-	-	-	32.0	-
Magazine	6.2	-	5.2	-	-	-	-	1.0	-
Social media	17.1	-	3.7	-	-	-	-	8.0	-
Retail outlets or pharmacies	31.9	-	26.2	-	-	-	-	4.0	-
**Received at least one**									
Sample of any baby milk product	4.7	10.0	10.9	1.2	14.0	1.2	14.2	3.0	7.4
Coupon of any baby milk product	2.9	13.0	1.6	0.3	1.5	0	6.7	1.2	3.4
Any gifts from someone other than a family member or a friend that may promote the use of a product covered^d^	34.1	17.6	8.5	2.7	24.8	1.5	16.0	6.9	14.0

^a^Disaggregated data from Ecuador, Nigeria, Panama, Philippines, and Thailand related to promotion outside health facilities are not included, as the reports used a different denominator than the total number of women

^b^Estimates totals are calculated only when all eight countries contributed data

^c^Includes maternity health facilities and public primary health facilities

^d^Includes bottles, diapers, clothing, breast shields, baskets, backpacks, utensils, pacifiers, toys, and checkbooks

**Fig. 1:**
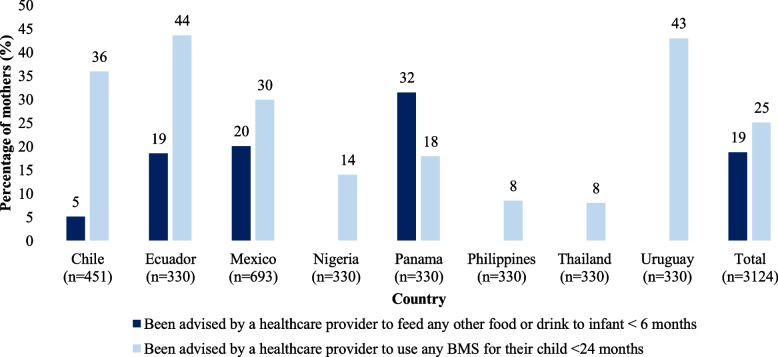
Mothers received advice from health care provider to feed food or drink to infant < 6 months or to use any breast-milk substitutes (BMS), by country^1^. *Note:* No information available for “Been advised by a health care provider to feed any other food or drink to infant < 6 months” for the Philippines, Nigeria, Thailand, and Uruguay. 1: Health care provider includes family/general doctor, nurse, gynecologist, midwife, pediatrician, and nutritionist

Nearly 20% of mothers with an infant younger than 6 months reported receiving advice from a health care provider to feed food or drink other than breast milk (Fig. 1). Infant formula was reported as most frequently recommended (data not shown). Twenty-five percent of mothers with a child younger than 24 months had been advised by a health care provider to use a BMS, in the previous 6 months; in four of the five countries surveyed in Latin America greater than 30% of these mothers were exposed to such messaging.

A total of 21% of health care providers reported having contact with a BMS representative in a health care facility in the previous 6 months (ranging from 2% in Nigeria to 53% in Panama) (Table 3). The most common contact was for distribution to mothers and other caregivers of a non-specified BMS sample (11%), promotional material (8%), or gifts (7%). Other reported reasons included offers of BMS for hospital use (11%) or an invitation to attend an event or workshop (8%).

**Table 3:** Health care providers who reported contact by representative of a breast-milk substitutes company in a health care facility, by country (%)

	**Chile (*n* = 164)**	**Ecuador (*n* = 66)**	**Mexico (*n* = 48)**	**Nigeria (*n* = 98)**	**Panama (*n* = 107)**	**Philippines (*n* = 126)**	**Thailand (*n* = 99)**	**Uruguay (*n* = 154)**	**Total**^**a**^** (*n* = 862)**
Contact with the health facility	43.3^b^	19.7	18.8	2.0	53.3	3.0	15.0	14.0	21.1
**Contact to provide for distribution to mothers and other caregivers**									
Promotional materials	25.6	-	2.1	-	5.6	2.4	-	5.8	8.3
Samples	32.3	1.5	-	2.0	18.7	3.0	15.0	4.6	11.0
Gifts	23.8	-	-	1.0	2.8	0.8	6.1	3.9	6.4
Coupons	-	-	6.3	-	0	0.8	-	0.6	1.9
**Contact to**									
Promotional materials for use of facilities/staff	-	-	10.4	-	18.7	-	-	2.6	10.6
Requests for display and other promotional activities in the facility	1.8	6.1	4.2	-	4.7	2.4	-	0.6	3.3
Seek direct contact with mothers and other caregivers	10	0	-	-	0.9	-	-	0	2.7
Make offers for free supplies of breast-milk substitutes	3.7	9.1	-	-	29.9	-	-	0	10.7
Distribute any other supply for the hospital use	-	15.2^c^	-	-	11.2^d^	-	-	1.3^e^	9.2
Received an invitation to attend an event/workshop outside the health facility by breast-milk substitute company	3.7	6.1	-	13.3	9.3	11.9	11.1	0	7.9

^a^Estimated totals and averages were obtained from available data

^b^Includes maternity health facilities and public primary health facilities

^c^Prevalence (%) of total health personnel who reported that baby food companies had contacted them to provide any item for the use in the facilities

^d^Prevalence (%) of total health personnel who reported that baby food companies had contacted them to sponsored activities or workshops

^e^Prevalence (%) of total health personnel who reported that baby food companies had contacted them to provide informational/educational materials

**Fig. 2:**
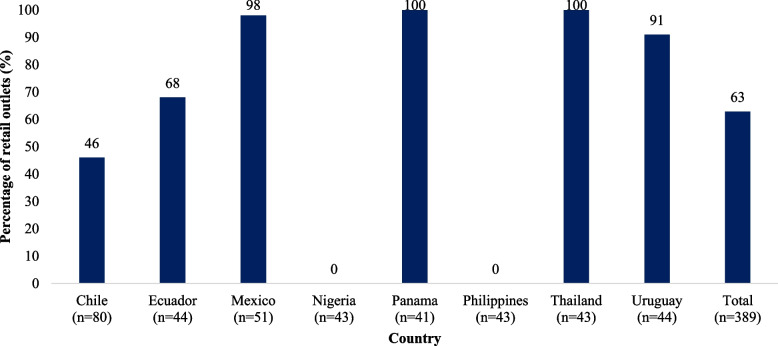
Promotions observed at retail outlets, by country

Of the 389 retail stores and pharmacies surveyed, promotions were observed in 63%, with a range from none in Nigeria and the Philippines to over 90% in Mexico, Panama, Thailand, and Uruguay (Fig. 2). In Mexico and Thailand, all or almost all retailers had promotions associated with products covered under the Code. Promotions about price reductions were most frequent (36%), followed by promotion in packages (18%) and gifts (10%) (data not shown).

**Fig. 3:**
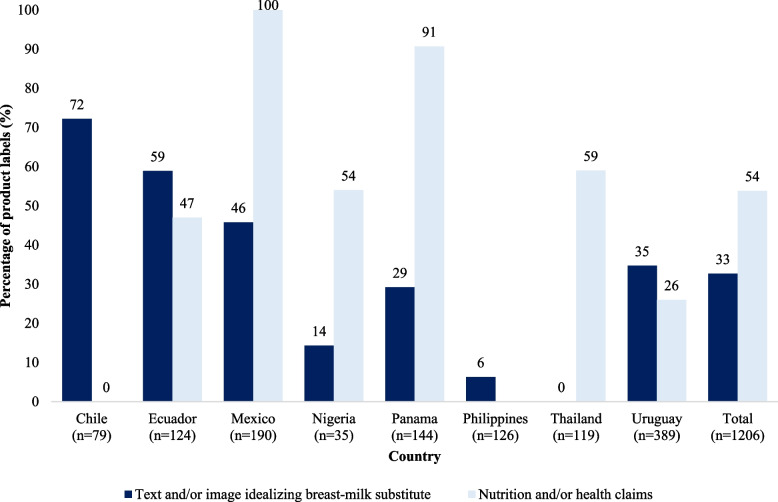
Product labels analysis, by country. *Note:* No information available about “Nutrition and/or health claims” for Philippines

Of the 1,206 labels and inserts of BMS products, infant formula and complementary foods comprised most of the sample. Nearly half of the labels included health and/or nutrition claims with large variation among countries (Fig. 3). For example, in Mexico all BMS products had such claims, whereas in Chile and the Philippines none had claims. Thirty-three percent of the labels included text or images that idealized bottle feeding, with Chile (72%) and Ecuador (59%) showing the highest prevalence. In countries where labels were disaggregated by type of formula (Chile, Ecuador, Panama, and the Philippines), the product types most non-compliant were complementary foods for children 6–35 months old in the Philippines and infant formula in Panama. In Chile, the special formulas (designed with specific modifications in their ingredients for sick infants) had the most violations followed by GUMs, and in Ecuador GUMS had the most violations (data not shown).

In Latin America, over 50% of health care providers reported no knowledge of the Code, and 50% reported no knowledge of national legislation. The highest percentage of health care providers reporting no knowledge of the Code or national legislation was in Chile, where 73% and 67% reported no knowledge of the Code or national legislation, respectively (Supplementary Fig. 1).

**Fig. 4:**
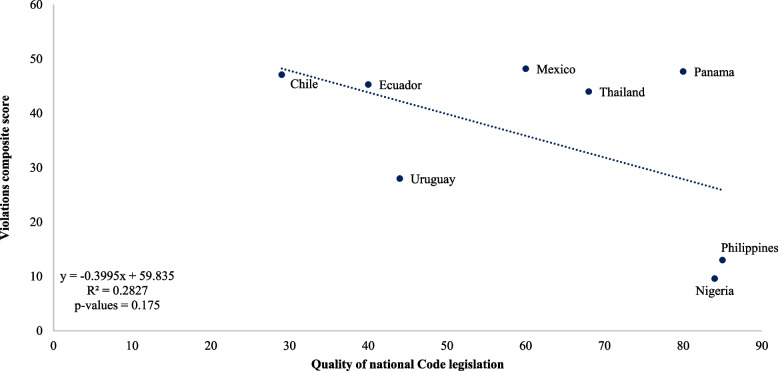
Violations composite score by alignment with the code, by country

A strong, though non-significant, linear relationship between the composite violations score and quality of Code legislation was found (Fig. 4). In general, countries with the lowest percentage of violations had the strongest Code legislation.

## Discussion

Our study shows that despite national legislation requiring a multibillion-dollar and growing global milk formula industry to account for its obligations under the Code, inappropriate marketing of BMS remains highly prevalent. Women reported exposure to BMS promotion from a variety of sources, including advertisement in traditional media, on the internet and social media, and in shops and pharmacies. Health care providers in all countries reported contact in a health care facility by a manufacturer or distributor of infant formula or other products covered under the Code. Our label analysis showed that health and/or nutritional claims or text and images idealizing BMS were common, again with great variation across countries.

According to the report of the National Implementation of the International Code, countries in our study show large variability in the extent to which Code provisions have been incorporated into law [6] (Supplemental Table 1). Chile, Ecuador, and Uruguay have the fewest incorporated provisions; Mexico and Thailand have the next most; and Nigeria, Panama, and the Philippines have the most provisions. In our analysis of Code violations against Code legislation, Mexico, Panama, and Thailand were notable outliers. In Mexico, while legislation is moderately aligned with the Code, the monitoring and enforcement component of the legislation score is quite low, suggesting that there may be more violations than expected because of poor enforcement. Anecdotal reports from Panama suggest that the high number of violations there may also be due to poor enforcement of existing legislation. In Thailand, the data were collected in January 2018, immediately after the Code legislation was adopted in 2017, and possibly indicate that the legislation had not yet had its intended effect.

Fewer violations were reported in Nigeria and the Philippines compared to other countries, a finding that may reflect the strong monitoring systems in place. Both countries had the lowest frequency of BMS promotion, particularly with respect to health care providers’ reported contacts from BMS companies; BMS promotions at retail stores and pharmacies; and health and nutrition claims, text, and images idealizing BMS on product labels. A potential explanation for the lack of such messaging idealizing BMS products may be that both countries have strong labeling provisions in their legislation. However, this is not the case in Thailand, which has strong monitoring and enforcement of the Code, but no provisions on labeling in its legislation. Also, even though all countries have provisions on promotions at point-of-sale retail outlets, except Chile and Ecuador, only in Nigeria and the Philippines were there no such BMS promotions, indicating that there is a lack of reinforcement of Code legislation with respect to this provision.

Our findings are consistent with evidence from other countries evaluating Code compliance. A study summarizing results from monitoring the Code in 10 countries from Asia and Africa compared the prevalence of country violations and relevant country legal measures [23]. Results like those in our study were reported, showing the existence of BMS promotion, recommendations to use BMS (ranging from 3% in Laos to 47% in Nepal), and provision of BMS sample gifts and/or coupons (up to 15%), both within and outside of the health care system. For promotion outside of the health care system, including advertisements and commercial and other materials through products, brands, and companies, the prevalence varied from 8% in Tanzania to 89% in Indonesia. Promotion from television was most common, ranging from 3% in Nepal to 85% in Indonesia. BMS promotions at point-of-sale in 8 of the 10 countries were reported, ranging from 3% in Nepal to 85% in Indonesia, and included gifts, reduced pricing, displays, and free samples, among other things. The use of pictures and text idealizing the use of BMS across countries was also reported.

A more recent multi-country study, commissioned by WHO and designed and implemented by M&C Saatchi World Services, collected data from eight countries across the WHO regions of Southeast Asia, Africa, the Americas, and Europe [24]. Like our study, the results show that marketing for formula milk is ubiquitous, through different channels, including traditional media, social networks, and digital media, as well as through health care providers. Our findings are also consistent with a recent scoping review that confirms that violations of the Code have not ceased [25]. This review also reported an increase in marketing through digital platforms and brand extensions.

Digital marketing of BMS is not only common, but particularly challenging to measure [26]. As with all digital marketing, messages about specific products can be tailored to individual consumers through multiple globally used platforms. These platforms can also be used to disseminate gifts, discounts, and coupons. Because these messages are directed to an individual, they cannot be monitored through traditional means. In Nigeria, for example, while the government is clearly effective in restricting promotions in traditional stores, action is needed to ensure its system structure to monitor promotions is expanded to also include online retailers as well [16].

Our paper has strengths; it is the first to analyze a set of studies that used the same WHO/UNICEF protocol and included countries from Africa, Asia, and Latin America. It also has its weaknesses. There were differences in implementation of the protocol. For example, in Chile private maternity wards were not sampled and in general there was no consistency in the proportion of public versus private maternity facilities sampled in the eight studies. Also, Nigeria, the Philippines, and Thailand used an abbreviated questionnaire for health care providers and mothers. These studies were also conducted by Westat, under contract with the Access to Nutrition Foundation, whereas the other five the studies were conducted by or with approval from national Ministries of Health, with technical support from UNICEF and or the Pan American Health Organization (PAHO). Marketing using digital platforms, which is increasing in frequency, is also difficult to measure and exposures from these sources are not well-reflected in our results.

The availability of a comprehensive WHO/UNICEF-endorsed protocol for measuring Code compliance provides a useful resource for cross-country comparisons. However, the cross-country comparisons we present in this paper were limited by the way the protocol was implemented and results were published in each country. Clearer guidance from WHO and UNICEF on standard output tables and graphs would aid comparability. As noted in Table 1 countries differed in the percentage of private versus public facilities surveyed and in the percentage of physicians, nurses, and obstetricians versus other clinic personal interviewed. Those implementing the NetCode Protocol should strive to achieve the recommended sample sizes. Lastly, the NetCode protocol provides only limited data on digital marketing of BMS. We encourage countries to explore innovative methodologies to capture digital promotions.

Breastfeeding requires successful initiation at birth, when mothers are particularly vulnerable to the influence from medical staff and hospital practices. Poor knowledge of the Code and national laws by health care providers, documented in our study, is deeply worrying, given the importance of health care providers as a source of information and support to mothers and families at this critical juncture in time [27].

## Conclusion

Our study, summarizing and comparing the results of recent monitoring of the Code in eight countries using the NetCode protocol, highlights three key facts: 1) the marketing of BMS is ubiquitous and multifaceted; 2) the high variability of promotion across countries generally reflects the comprehensiveness of Code legislation; and 3) health care providers exhibit poor knowledge of the Code and national legislation. These findings call for several policy actions, including strengthening and enforcing national Code legislation and development and implementation of ongoing monitoring mechanisms at the national level, including in the digital space. Action should also include promoting information on the Code and responsibilities of health care providers under the Code in relevant preservice and in-service training curricula, symposia, and conferences.

## Supplementary Information

**Additional file 1: Supplementary Table 1.** Country World Bank income classification, score, and degree of alignment with the Code. **Supplementary Table 2.** Characteristics and methodological differences between the reports included in the present study. **Supplementary Table 3.** Channels of breast-milk substitute promotion and weight distributions used to construct composite score of violations. **Supplementary Figure 1.** Health providers who refer no knowledge of the Code nor national laws or standards related to it, by country.

## Data Availability

The data presented in this study are available on request from the corresponding author.
