# Supplementary material for: Violations of the International Code of Marketing of Breast-milk Substitutes: a multi-country analysis
Source: BMC Public Health. 2022 Dec 13;22:2336. doi: 10.1186/s12889-022-14503-z (PMC9749209; doi:10.1186/s12889-022-14503-z)
Supplement: Supplementary file 1 — Additional file 1: Supplementary Table 1. Country World Bank income classification, score, and degree of alignment with the Code. Supplementary Table 2. Characteristics and methodological differences between the reports included in the present study. Supplementary Table 3. Channels of breast-milk substitute promotion and weight distributions used to construct composite score of violations. Supplementary Figure 1. Health providers who refer no knowledge of the Code nor national laws or standards related to it, by country. [file 12889_2022_14503_MOESM1_ESM.docx]

**Supplementary Table 1.** Country World Bank income classification, score, and degree of alignment with the Code

| **Country** | **World Bank income classification** | **Total score (out of 100)** | **Degree of alignment** |
| --- | --- | --- | --- |
| Chile | Upper income | 29 | Some provisions included |
| Ecuador | Upper-middle income | 40 | Some provisions included |
| Mexico | Upper-middle income | 60 | Moderately aligned |
| Nigeria | Lower-middle income | 84 | Substantially aligned |
| Panama | Upper-middle income | 80 | Substantially aligned |
| Philippines | Lower-middle income | 85 | Substantially aligned |
| Thailand | Upper-middle income | 65 | Moderately aligned |
| Uruguay | Upper income | 47 | Some provisions included |

Source: World Bank. *New World Bank country classifications by income level: 2022-2023*. 2022 [cited 2022 August 11]; Available from: <https://blogs.worldbank.org/opendata/new-world-bank-country-classifications-income-level-2022-2023>

Supplementary Table 2. Characteristics and methodological differences between the reports included in the present study

|  | **Chile** | **Ecuador** | **Mexico** | **Nigeria** | **Panama** | **Philippines** | **Thailand** | **Uruguay** |
| --- | --- | --- | --- | --- | --- | --- | --- | --- |
| City | Santiago | Quito and Guayaquil | Chihuahua and Puebla | Lagos | San Miguelito and Northern Panama | National Capital Region | Bangkok | Montevideo |
| Publication date | December 2017 | March 2017 | March 2016 | April 2018 | October 2019 | March 2021 | January 2018 | August 2019 |
| NetCode protocol version | 2015^1^ | 2015 | 2015 | 2015^1^ | 2018 | 2018 | 2015^1^ | 2018 |
| Primary objective | Analyze violations of the International Code of BMS. | Estimate compliance by health establishments and points of sale. | Estimate compliance and to measure the scale of violations by health facilities. | Monitor compliance by all manufacturers selling breast-milk substitute (BMS) products. | To evaluate promotional practices of manufacturers selling BMS products, bottles, and teats. | Monitor compliance by all manufacturers selling BMS products. | Monitor compliance by all manufacturers selling BMS products. | Evaluate the level of compliance with the provisions of the Code. |
| Women included in the study | Convenience and simple random sampling. | Some homes near the health facility were visited.^2^ Convenience sampling. | Convenience sampling and computer‐automated sampling method. | Convenience sampling. | Stratified sampling. | Some homes near the health facility were visited.^2^ Convenience sampling. | Convenience sampling. | Probability sampling. |
| Health providers included in the study | Nutritionists in charge of the Milk Dietetic Service were included. Convenience sampling. | Convenience sampling. | Convenience sampling. | Convenience sampling. | Convenience sampling. | Convenience sampling. | Convenience sampling. | Probability sampling. |
| Health facilities included in the study | Only public health facilities were included. Convenience sampling. | Random sampling. | Doctor´s office was included.^2^ Random sampling (public facilities) and convenience sampling (private facilities). | Maternity facilities were not included. Random sampling. | Probability proportional to size systematic sampling. | Probability proportional to size systematic sampling. | Probability proportional to size systematic sampling. | Maternity facilities were not included. Probability proportional to size systematic sampling. |
| Retail outlets included in the study | Convenience sampling. Online retail sites were included. | Convenience sampling. | Convenience sampling. | Convenience sampling. Online retail sites were included. | Convenience sampling. Online retail sites were included. | Convenience sampling. Online retail sites were included. | Convenience sampling. Online retail sites were included. | Convenience sampling. Online retail sites were included. |
| BMS products included in the analysis | BMS products for children < 36 months. Bottles, pacifiers, and teats were included. | BMS products for children < 24 months. Bottles, pacifiers, and teats were included. | BMS products for children < 24 months were included. Bottles, pacifiers, and teats were not included. | BMS products for children < 36 months were included. Bottles, pacifiers, and teats were not included. | BMS products for children < 36 months were included. Bottles and teats were included. | BMS products for children < 36 months were included. Bottles, pacifiers, and teats were not included | BMS products for children < 36 months were included. Bottles, pacifiers, and teats were not included | BMS products for children < 36 months. Bottles, pacifiers, and teats were included. |
| Media monitoring^3^ | NA (however, they assessed TV and internet). | NA | NA | NA (however, they assessed TV and internet). | TV and internet advertising were evaluated. | TV and internet advertising were evaluated. | NA (however, they assessed TV and internet) | TV advertisements were not evaluated. |

NA: Not Applicable.

1: Includes the recommendations of the World Health Assembly Resolution 69.9: Marketing and promotion on online retail sites and websites evaluation.

2: If the sample was not completed during the visit to the health facility, some women who were regular clients of the health facility were interviewed at their homes.

3: Media component was included until the 2017 NetCode version. Two elements are assessed separately: television and internet advertising.

**Supplementary Table 3.** Channels of breast-milk substitute promotion and weight distributions used to construct composite score of violations

| **Channels of BMS promotion and subtypes** | **Points** |
| --- | --- |
| Mothers’ exposure to promotions (%) | 40 |
| Exposed to promotion in general | 25 |
| Receiving a sample | 5 |
| Receiving a coupon | 5 |
| Receiving other product-related gifts | 5 |
| Promotion through health care providers (%) | 20 |
| Receiving a promotional material for mothers | 5 |
| Receiving a sample for mothers | 5 |
| Receiving a gift for mothers | 5 |
| Receiving a coupon for mothers | 5 |
| Promotions in retail outlets (%) | 20 |
| With a visible promotion | 20 |
| Promotions on product labels (%) | 20 |
| With idealizing imagery | 10 |
| With health and/or nutrition claims | 10 |
| Total points | 100 |

Supplementary Figure 1. Health providers who refer no knowledge of the Code nor national laws or standards related to it, by country


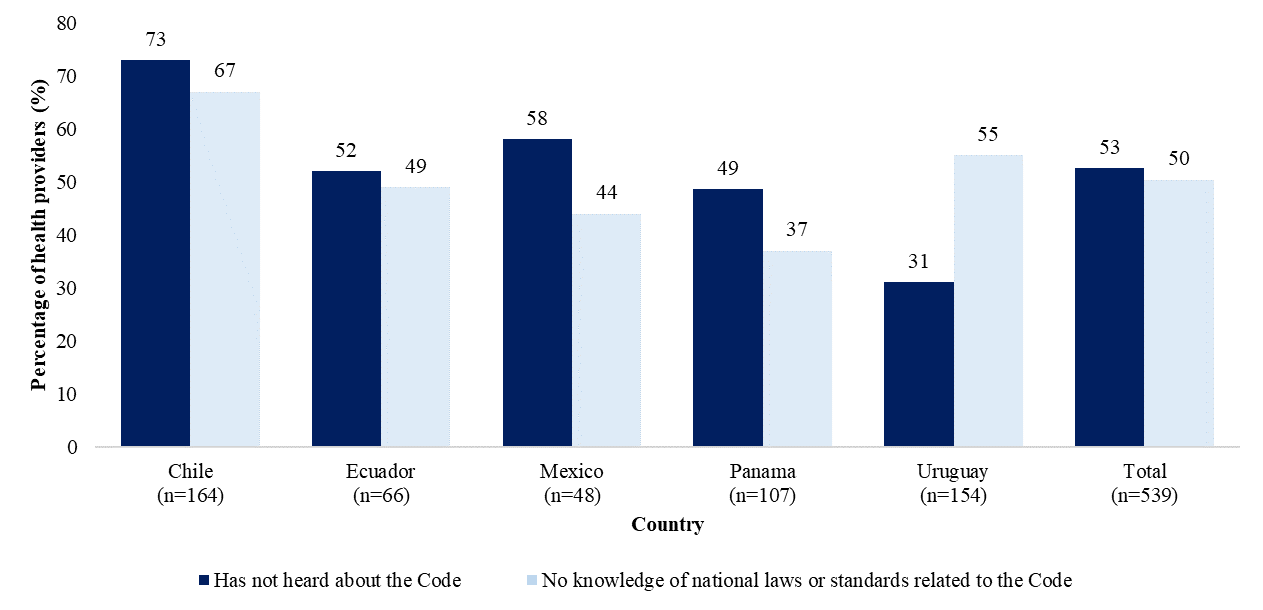


*Note:* No information available for Nigeria, the Philippines, and Thailand.
